# Brachial Plexus Block Following Serratus Anterior Plane Block After Minimally Invasive Cardiac Surgery Identified Using Contrast X‐Ray: A Case Report

**DOI:** 10.1155/cria/9755915

**Published:** 2026-01-06

**Authors:** Yuna Sato, Michio Kumagai, Yusuke Takei, Kazutomo Saito, Takahiro Tasaki, Masanori Yamauchi

**Affiliations:** ^1^ Department of Anesthesiology and Perioperative Medicine, Tohoku University Graduate School of Medicine, Sendai, Japan, tohoku.ac.jp; ^2^ Division of Anesthesia, Japanese Red Cross Ishinomaki Hospital, Ishinomaki, Japan, jrc.or.jp; ^3^ Department of Anesthesiology, Sendai Kousei Hospital, Sendai, Japan, sendai-kousei-hospital.jp

**Keywords:** brachial plexus block, case report, minimally invasive cardiac surgery, peripheral nerve block, serratus anterior plane block

## Abstract

A serratus anterior plane block is effective for pain management after minimally invasive cardiac surgery (MICS) but may cause rare complications. In this study, we present a case of upper limb neurological symptoms following a serratus anterior plane block (SAPB) administered after MICS, which was visualized via contrast x‐ray imaging. A 74‐year‐old woman with severe aortic stenosis underwent aortic valve replacement via minithoracotomy. We preoperatively inserted a SAPB catheter deep into the serratus anterior muscle (SAM). Postoperative x‐ray imaging with contrast medium revealed its distribution extending from the chest wall (second to fourth ribs), detached from the thorax, toward the right upper arm. We initiated a programmed intermittent bolus infusion (PIBI) via the catheter, but the patient developed reduced grip and sensory deficits in the right arm. Discontinuation of PIBI on postoperative day five led to neurological symptom resolution by day six. The clinical course and contrast distribution suggested that the catheter tip may have migrated to the superficial layer of the SAM. These findings highlight the importance of confirming catheter placement and carefully monitoring neurological symptoms when atypical contrast spread patterns are observed.

## 1. Introduction

Minimally invasive cardiac surgery (MICS) typically involves a small lateral thoracotomy and is associated with fewer complications than traditional medial sternotomy [1]. Nevertheless, damage to the thoracic wall muscles, ribs, or intercostal nerves may result in post‐MICS pain [2].

Serratus anterior plane block (SAPB) has the potential to improve post‐MICS pain management by blocking the lateral cutaneous branches of the intercostal nerves, which innervate the lateral chest wall [3]. Although the effectiveness of continuous SAPB in post‐MICS analgesia has been demonstrated, reports on block‐related complications remain limited [4–6].

Herein, we report a rare and educational case of upper extremity neurological symptoms, presumed to be related to continuous SAPB for post‐MICS analgesia, with contrast‐enhanced radiography aiding in the diagnosis.

## 2. Case Presentation

A 74‐year‐old woman (height: 150 cm and weight: 43 kg) with severe aortic stenosis (AS) underwent MICS with aortic valve replacement (AVR) via a right‐sided minithoracotomy. The patient was diagnosed with moderate AS at the age of 72 years, which progressed to severe AS. Following confirmation of normal renal function and absence of contrast allergy, the use of a SAPB catheter and the imaging evaluation protocol were explained to the patient, and written informed consent was obtained.

General anesthesia was induced with midazolam (2 mg), remifentanil (total bolus: 100 μg), and rocuronium (50 mg) and maintained with total intravenous propofol infusion. SAPB was performed in the supine position with internal rotation of the right brachium. A 6–13‐MHz linear probe (SonoSite S II; FUJIFILM Medical Co., Tokyo, Japan) was placed on the fourth rib near the middle axillary line in the sagittal plane. A 17‐gauge Tuohy needle (Hakko Co., Nagano, Japan) was advanced onto the rib, and 15 mL of 0.375% ropivacaine was administered, hydrolocating the fascial plane between the serratus anterior muscle (SAM) and the rib. A multiple‐hole catheter (Hakko Co.) was inserted 20 cm into the hydrolocated fascial plane, targeting the deep layer of the SAM, followed by an additional 15 mL of 0.375% ropivacaine. MICS AVR was performed via the third intercostal space. Cardiopulmonary bypass (CPB) was established via the right femoral artery and vein. As an abdominal aortic thrombus was present, a prosthetic vascular graft was anastomosed to the right axillary artery and used as an additional arterial inflow route to prevent retrograde embolization. The surgical course was uneventful.

**Figure 1:**
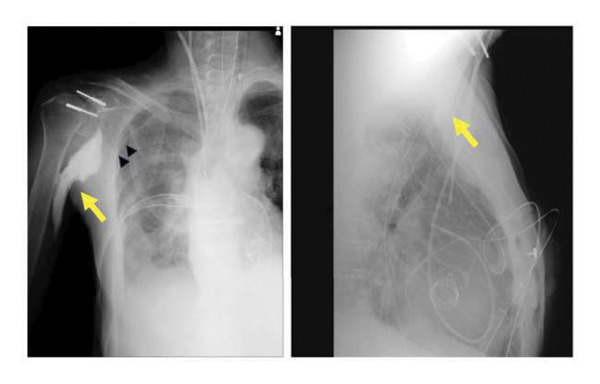
Postoperative frontal and lateral chest radiographs showing contrast medium spreading from the serratus anterior plane block catheter. The contrast medium (yellow arrow) is seen extending from the chest wall (second to fourth ribs), detached from the thorax, toward the right brachium. The fourth rib is marked with black arrowheads.

At the end of surgery, 10 mL each of iohexol (Omnipaque 240, GE Healthcare, Chicago, IL, USA) and 0.75% ropivacaine were administered via the SAPB catheter to evaluate anesthetic spread and confirm catheter placement. Radiographic imaging demonstrated contrast distribution extending from the chest wall at the second to fourth rib levels, detached from the thoracic cavity, to the right upper arm (Figure 1). After admission to the intensive care unit, a programmed intermittent bolus infusion (PIBI) pump (CADD‐Legacy PLUS; Smiths Medical, St. Paul, MN, USA) was connected to the SAPB catheter to deliver 15 mL of 0.25% levobupivacaine every 4 h.

The patient was extubated on the morning of postoperative day (POD) 1, at which point she voluntarily reported decreased grip strength in the right upper extremity and numbness in the second through fifth digits. The patient experienced hypoesthesia in the second and third digits, as well as in the right forearm, accompanied by hypalgesia in the same forearm. Motor function, including flexion and extension of the right wrist, elbow, and shoulder joints, remained intact. The distribution of neurological symptoms suggested involvement of the C7‐C8 dermatomes and possibly the medial cord of the brachial plexus, originating from the lower trunk. We initially considered these symptoms to be related to brachial plexopathy caused by surgical manipulation around the axillary artery and brachial plexus, and oral mecobalamin was administered. The numerical rating scale score remained zero, except for a transient increase to five with movement on POD 2, which was relieved by acetaminophen administration. At the request of the patient, PIBI was continued for 5 days. The SAPB catheter was removed on POD 5, and all neurological symptoms except numbness in the second through fifth digits resolved by the following day. The patient was discharged on POD 17 with mild residual numbness.

## 3. Discussion

SAPB can be performed in either the superficial or deep layer of the SAM, with studies demonstrating comparable efficacy for both approaches [3]. In our previous randomized controlled trial, we evaluated the analgesic efficacy and systemic safety of SAPB with PIBI in MICS patients using serum ropivacaine concentrations and found it to be safe and well tolerated [7]. In the present case, postoperative analgesia was achieved via PIBI of SAPB, consistent with previous reports [4–7]. However, neurological symptoms involving the ulnar, medial, and medial cutaneous nerves of the forearm were also observed. This case serves as a cautionary example that although SAPB is generally considered safe, it may be associated with unintended spread to the brachial plexus.

**Figure 2:**
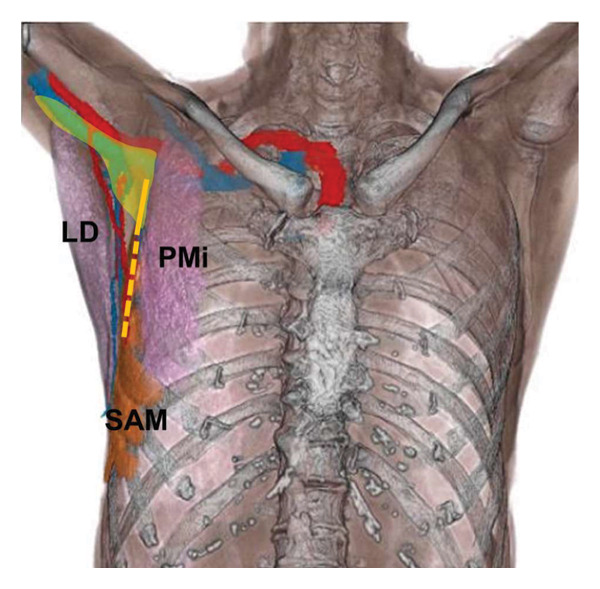
Anatomical relationships of the serratus anterior muscle and surrounding structures reconstructed from the patient’s CT scan. The yellow area shows image of the postoperative spread of contrast medium. The presumed the serratus anterior plane block (SAPB) catheter pathway is shown in orange dashed lines (deep serratus anterior muscle (SAM) layer) and solid lines (migrated to superficial layer). The local anesthetic administered via the SAPB catheter is thought to have spread from the superficial layer of the SAM into the costoclavicular‐SAM space, potentially affecting the medial cord of the brachial plexus. SAM, serratus anterior muscle; PMi, pectoralis minor muscle; LD, latissimus dorsi.

Postoperative radiography demonstrated contrast medium extending from the chest wall, at the second to fourth rib level, detached from the thorax to the right upper arm. This pattern of contrast distribution indicated that the tip of the SAPB catheter had migrated from the deep layer of the SAM, where the catheter had initially been placed under ultrasound guidance. Anatomically, the surface of the SAM at the second rib level corresponds to the costoclavicular‐SAM space, which communicates with the brachial plexus [8]. This anatomical relationship suggested that the tip of the SAPB catheter may have migrated into this space, allowing the local anesthetic to spread along the brachial plexus, resulting in the observed neurological symptoms (Figure 2). The medial cord of the brachial plexus was located at the nearest site to this space, where the local anesthetic was likely distributed. Grip weakness and sensory loss from the forearm to the second and third digits corresponded to the distribution of the ulnar, median, and medial cutaneous nerves of the forearm, suggesting involvement of the medial cord of the brachial plexus [9]. Catheter tip migration may have been facilitated by the 20‐cm insertion depth, particularly in a small patient with diffuse muscle atrophy. At our institution, we routinely insert the catheter to 20 cm to ensure that all orifices of the multiple‐hole catheter remain within the target plane and to reduce the risk of backflow. However, in retrospect, especially in small patients, more cautious selection of catheter insertion depth could help minimize the risk of migration. The insertion depth could be determined by adding the ultrasound‐measured distance from the skin to the target fascial plane to the length from the catheter tip to the most proximal orifice. Additionally, real‐time ultrasound confirmation of the final catheter tip position may help detect inadvertent deviation from the intended plane. Deep catheter insertion can increase the risk of complications, such as exposure of the catheter in the surgical field and mechanical damage or kinking caused by surgical manipulation. Additionally, the surgical procedure for CPB was performed in the same anatomical region. Although the surgical team did not observe the catheter tip or local anesthetic intraoperatively, the possibility of surgical manipulation contributing to catheter displacement or nerve irritation cannot be excluded. The paralysis resolved after discontinuation of PIBI for SAPB, except from residual numbness in the digits. These factors may have allowed the catheter tip to migrate into the costoclavicular‐SAM space.

Despite the adverse effects of SAPB on the upper extremities, we continued PIBI with SAPB for two critical reasons. First, superficial SAPB provided effective postoperative analgesia in this patient, radiographic imaging demonstrated sufficient anesthetic distribution at the surgical site, and the patient was satisfied with the treatment. Second, the neurological symptoms of the right upper limb observed in this patient could be explained by a brachial plexus injury resulting from axillary artery cannulation for CPB, which reportedly occurs in 0.7%–2.9% of the cases [10, 11]. Initially, we considered the symptoms to be a consequence of surgical manipulation; therefore, rather than discontinuing PIBI for diagnostic purposes, we decided to continue with the infusion. Additionally, we administered oral mecobalamin and monitored the patient’s neurological symptoms over time. The clinical course of the patient suggested that the axillary artery cannulation probably caused the persistent numbness in the digits until discharge.

In conclusion, we report a case of upper limb neurological symptoms associated with SAPB via catheter, supported by postoperative radiographic imaging with contrast medium and present the clinical course. This case emphasizes the importance of confirming catheter position and the spread of local anesthetic when performing SAPB catheter insertion. In retrospect, this complication might have been avoided if we had more carefully considered the size of the patient body and muscle atrophy and adjusted the catheter insertion depth accordingly. Additionally, although the contrast spread pattern was initially interpreted as acceptable for SAPB, its atypical features should have prompted consideration of potential brachial plexus involvement when neurological symptoms emerged.

This rare case of upper limb neuropathy caused by unintended spread of local anesthetics to the brachial plexus via an SAPB catheter highlights the importance of careful catheter placement and interpretation of imaging findings. Future investigations into catheter placement strategies tailored to different body types, as well as studies exploring the variability of contrast spread patterns in SAPB with catheter use, may help reduce the risk of similar complications.

## Consent

All the patients allowed personal data processing, and informed consent was obtained from all individual participants included in the study.

## Conflicts of Interest

The authors declare no conflicts of interest.

## Author Contributions

Yuna Sato: conceptualization, interpretation, and manuscript drafting. Michio Kumagai, Yusuke Takei, Kazutomo Saito, Takahiro Tasaki, and Masanori Yamauchi: clinical discussion, case interpretation, and critical manuscript revision.

## Funding

This work was supported by JSPS KAKENHI (grant number JP25K19826 to Yuna Sato).

## Data Availability

The data that support the findings of this study are available from the corresponding author upon reasonable request.
